# Causes of adverse events in home mechanical ventilation: a nursing perspective

**DOI:** 10.1186/s12912-022-01038-2

**Published:** 2022-09-27

**Authors:** Myriam Lipprandt, Wenke Liedtke, Martin Langanke, Andrea Klausen, Nicole Baumgarten, Rainer Röhrig

**Affiliations:** 1 Institute of Medical Informatics, Medical Faculty, RWTH Aachen University, Aachen, Germany; 2 Protestant University of Applied Sciences, Bochum, Germany; 3 Carl von Ossietzky University of Oldenburg, Oldenburg, Germany; 4 University of Sheffield, School of Languages and Cultures, Sheffield, UK

**Keywords:** Patient safety, Mechanical ventilators, Adverse events, Home nursing, Risk management

## Abstract

**Background:**

Adverse events (AE) are ubiquitous in home mechanical ventilation (HMV) and can jeopardise patient safety. One particular source of error is human interaction with life-sustaining medical devices, such as the ventilator. The objective is to understand these errors and to be able to take appropriate action. With a systematic analysis of the hazards associated with HMV and their causes, measures can be taken to prevent damage to patient health.

**Methods:**

A systematic adverse events analysis process was conducted to identify the causes of AE in intensive home care. The analysis process consisted of three steps. 1) An input phase consisting of an expert interview and a questionnaire. 2) Analysis and categorisation of the data into a root-cause diagram to help identify the causes of AE. 3) Derivation of risk mitigation measures to help avoid AE.

**Results:**

The nursing staff reported that patient transportation, suction and tracheostomy decannulation were the main factors that cause AE. They would welcome support measures such as checklists for care activities and a reminder function, for e.g. tube changes. Risk mitigation measures are given for many of the causes listed in the root-cause diagram. These include measures such as device and care competence, as well as improvements to be made by the equipment providers and manufacturers. The first step in addressing AE is transparency and an open approach to errors and near misses. A systematic error analysis can prevent patient harm through a preventive approach.

**Conclusion:**

Risks in HMV were identified based on a qualitative approach. The collected data was systematically mapped onto a root-cause diagram. Using the root-cause diagram, some of the causes were analysed for risk mitigation. For manufacturers, caregivers and care services requirements for intervention offers the possibility to create a checklist for particularly risky care activities.

## Background

Home mechanical ventilation (HMV) is an established method of treating patients with lung failure caused by either chronic obstructive pulmonary disease or neurological disease [1]. In cases when mask ventilation is not possible or is insufficient, the patient can be given invasive ventilation via a tracheostoma and tracheostomy tube. Permanently ventilated patients live at home or in long-term care facilities. They are cared for by nursing staff, sometimes 24/7. Given that there are no national registers, it is not possible to state the exact number of patients receiving HMV [2]. According to an estimate, about 6.5 per 100,000 population were in receipt of HMV in 2001, of which about 12% were ventilated invasively [3]. There are large, national differences in HMV. A comparison of European epidemiological data shows that 6.6 patients per 100,000 population are ventilated out-of-hospital [4]. It is to be expected that demographic changes and the COVID-19 pandemic have led to an increase in the number of HMV patients. Neither reliable epidemiological data nor national registers exist at present.

Due to the specific requirements profile, nursing professionals in Germany must be certified and undergo advanced training to become an expert in HMV nursing. They are expected to have specialist knowledge in respiratory physiology and ventilation, ventilator technology, tracheostoma management, methods of secretion mobilisation and elimination, as well as crisis management/emergency management [5].

Caring for patients who are dependent on a life-sustaining ventilator is a challenging activity, which places enormous responsibility on caregivers. Emergencies (e.g. acute exacerbation or obstruction of the respiratory system) caused by failure, malfunction or improper use of the ventilator can lead to irreversible brain damage or death within minutes. Furthermore, the caregiver can become a “second victim” of the incident [6]. These critical incidents [7] are common in HMV and require immediate action by the caregiver [8] without admitting patients to hospital for unnecessary and sometimes undesired treatment [9]. The caregiver has to assess the severity of the incident and is responsible for taking decisions (e.g. consulting a doctor or making an emergency call). Thus, an “incident during care that results in patient harm” is defined as an adverse event (AE) [10]. These incidents are ubiquitous in a medical and nursing context and can lead to patient harm [11, 12]. AE often occur in the form of medication errors, infections, falls, and incorrect or delayed diagnoses [10], but technology-related events are also mentioned in the literature [13–15]. It is often the case that no distinction is made between the consequences and the harm resulting from an AE [13]. The need for action under intense pressure can lead to stress. This can undermine the caregiver’s ability to make considered judgements and can lead to wrong decisions [16], which can trigger AE [12, 17].

In a clinical setting, data on AE is mostly collected through studies or reporting systems. By contrast, the data that exists on AE occurring in a home care setting is very poor [13, 18]. There is little data available on ventilator errors and the consequences for patients, especially in HMV, as there are no national registers [2]. There are no established reporting systems, nor is there a legal requirement for a structured error-handling framework. The circumstances in which AE occur in a home setting cannot be compared to those in a clinical setting. Despite underreporting, it can be assumed that AE that result in patient harm also occur in an outpatient setting [13, 18].

Therefore, carrying out an analysis of near misses and AE can help to better understand the dynamics of AE in general [14, 19, 20]. In the systematic analysis of AE, it is assumed that the incident is always preceded by a chain of multiple causes [21]. It is essential to understand the individual links (causes and contributing factors) in the chain of causes to be able to break the links and prevent future incidents and risks. Therefore, all risks must first be identified to ensure safety in a clinical and nursing context [11]. Risk management [19, 22] is also crucial for nursing activities, especially when they involve medical devices. The objective is therefore to prevent patient harm by learning from AE and their causes.

The joint project Mesib (https://www.mesib.de/) aimed to improve the safety of patients in HMV. By developing a safety-critical IT infrastructure, it should be possible to preventively mitigate critical emergencies. The results of the work described here serve as an extended requirements analysis and identification of emergencies in HMV for the technology development in Mesib.

The aim of this study is to identify the nursing activities that can lead to AE in HMV. Based on this, a model describing the causes of adverse events has been developed and can serve as a basis for a practice-related safety concept for HMV. A combined methods approach involving intensive care practitioners in hospital and home care was used. The study concludes with a list of recommended actions for manufacturers of respiratory equipment, identifies further training requirements for nursing staff, and suggests various organisational measures for nursing services.

## Methods

### Design

**Fig. 1:**
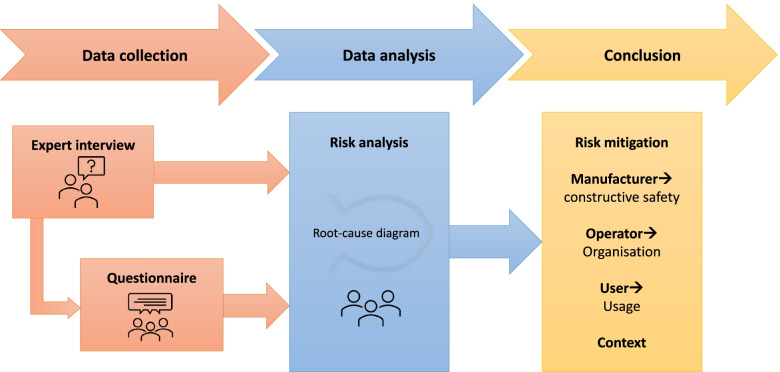
Extended mixed-method approach

A multi-level data collection process was conducted involving expert interviews and a questionnaire. The qualitative results were analysed using systematic error analysis methods (root-cause diagram) with information and knowledge on technology and medicine. This made it possible to identify the causal, requisite and contributing factors that lead to critical situations and patient harm, i.e. AE. In the application area of medical software engineering, or health informatics and nursing informatics, it is essential to always consider the entire socio-technical system. Errors can occur as a result of, for example, the product (manufacturer responsibility), operation (organisational responsibility), operation (user), communication (team), or an unforeseeable situation. The categories (bones) of the Ishikawa diagram (chapter 2.3.2) were adapted to the language and concepts of carers for better comprehensibility (see Fig. 1).

The risk factors identified through the root-cause diagram form the basis for the development of risk mitigation measures. These measures should be implemented across all levels of the socio-technical system and within the various areas of responsibility. A number of example measures were derived from the results in order to demonstrate the wide spectrum of possible measures and options for action at all levels of the socio-technical system. These measures were developed by an interdisciplinary team consisting of nurses, medical informatic scientists, medical professionals (anaesthetists), ethicians, and social scientists.

### Participants

The participants of the study were employees of institutions that were project partners of the Mesib research project. Due to the difficulty in recruiting participants because of staff shortages, hospital and nursing staff allowed participants to attend the interviews and answer the questionnaire during working hours. This increased participant willingness to partake in the study as it did not take up any of their own free time. Participation was completely voluntary. The inclusion criterion was based on roles in the facilities (hospital and nursing service). Given that only registered nurses are allowed to work in nursing, this was the inclusion criterion.

#### Expert interview

Expert interviews are a frequently used method in the information sciences; they are also used for knowledge modelling [23, 24]. We used this method to obtain initial information about processes and events in everyday nursing and clinical practice, with the aim of identifying the causes of risks from everyday practice. We interviewed two experts who are active in the training of nurses, work as nursing department heads, and have many years of professional experience in nursing themselves. The experts had a lot of inherent knowledge about the domain and were representative of the group of nursing experts. The experts were asked to list all of the activities that could involve medical device interaction and the unforeseen events that have occurred. Due to the overlap in results between the two interviews and thus reaching data saturation point, no further interviews were necessary, especially because the results adequately described the domain [25]. The interviewers were an anaesthetist and a computer scientist who had expertise on ventilators and human-machine interaction.

#### Questionnaire

Next, an online questionnaire was given to a small group of professional caregivers: *n*=11 participants in total (six nurses for intensive care weaning units and five outpatient nursing staff form an outpatient intensive care service located in Oldenburg, Germany).

The care tasks that formed the subject of the questions in the questionnaire were based on the expert interviews.

The questionnaire consisted of two qualitative and two quantitative questions. The aim was to validate the interview results, to collect further examples of AE and risk-related nursing activities from individual nursing experiences, and to identify potential similarities across a larger set of professionals. This data is not quantitatively useful for statistical purposes, but it does reflect a tendency towards risk-relatedness in two institutions and a strong need for support provision.

### Data analysis

#### Expert interview

The result of the interviews consisted of a list of activities involving device interaction (e.g. suction) and the unforeseen events (e.g. vomiting). In this respect, the interview formed part of the initial evaluation process, with a topic being analysed in collaboration with the experts. Following the expert interviews, the data was summarised and classified into categories according to the Ishikawa model [26] adapted by Canham et al. [27]. However, only the unforeseen events were listed, not the nursing activities, as these were used again in the questionnaire.

#### Analysing adverse events in HMV

The data was collected, analysed and interpreted by a multidisciplinary team consisting of nurses, medical informatic scientists, medical professionals (anaesthetists), ethicians, and social scientists.

The causes of AE were grouped into an Ishikawa diagram (*root-cause diagram*) [26] with categories adapted to those of Canham et al. [27]. Ishikawa diagrams are typically used in product design and quality management, but also in systematic failure analysis, where they help to identify and categorise contributing factors and causes of failure [28, 29]. The data of the analysis was extended to include the guidelines for mechanical ventilation [5].

#### Recommendations for risk mitigation

Based on the categorisation of the root-cause diagram, requirements for intervention can be derived for nurses, care providers and manufacturers of ventilators and equipment/supplies. The requirements for patient safety derive from risk management according to ISO 14971 and the London Protocol [30].

## Results

### Expert interviews

The findings from the expert interviews enabled the formulation of a list of adverse event causes. The causes that were found to occur during nursing care are presented in Table 1. The sequence of the items in the tables is random, but the list is grouped into the main categories of the root-cause diagram.

**Table 1:** Potential causes of AE during nursing activities grouped into the main categories of the root-cause diagram

Category (Ishikawa)	Causes of adverse events in HMV
Patient (health-related circumstances)	Heart frequency reduction (vagus stimulus)
	Vomiting
	Aspiration
	Hyperventilation
	Tracheal ulcer
	Pneumonia
	Health deterioration
	Swallowing disorders
	Diffusion disorders
	Compliance disorder
	(Airway) obstruction
	Drug reaction (tachycardia)
	Swelling of the respiratory tract
Machine and Material	Bending of the breathing tube
	Oxygen leakage, leakage in the cuff
	Extubation, tracheostomy decannulation, relocation
	Leakage in the hose system
	Device faults, power blackout
	Ventilation filter obstructed
Caregiver	Caregiver asleep during night

### Questionnaires

#### Q1: frequency of occurrence of adverse events during nursing activities

**Fig. 2:**
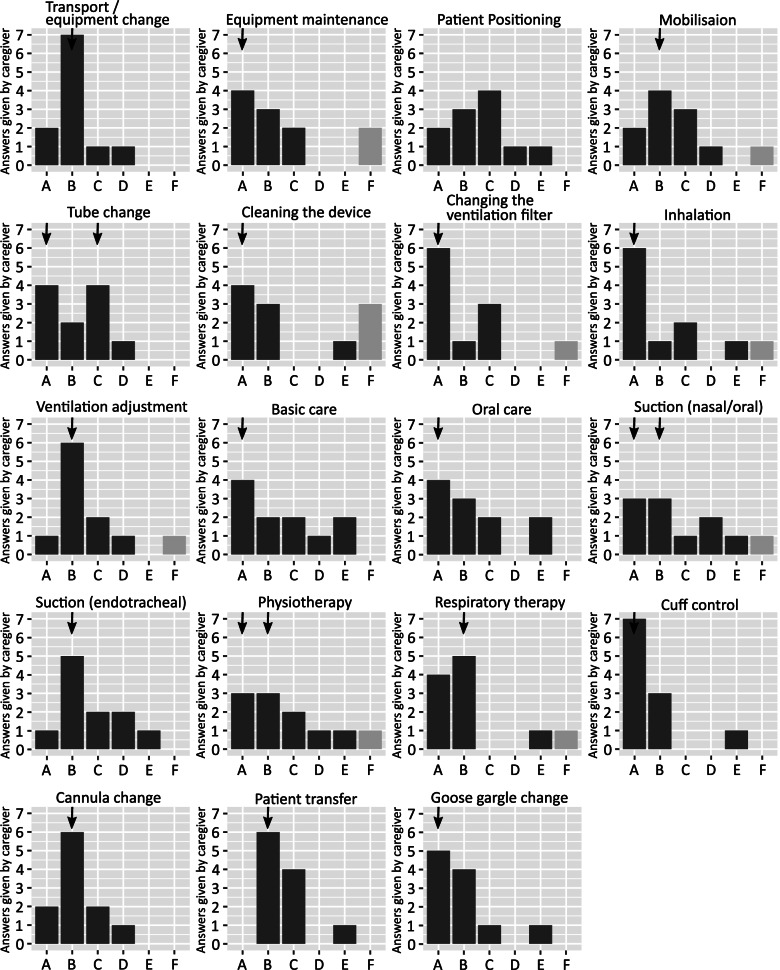
Shows the various care activities and the frequency with which AE can occur for each activity. A = never”, “B=1 x 3 month”, “C=1 x month”, “D=every 14 days”, “E = daily”, “F = cannot say”

The nurses were asked to indicate how often critical events occur when carrying out the 19 listed nursing activities (e.g. suction, equipment maintenance, patient transfer). The nurses (*n* = 11) were asked to categorise the nursing activities according to their criticality as “A = never”, “B=1 x 3 month”, “C=1 x month”, “D=every 14 days”, “E = daily”, “F = cannot say” (see Fig. 2).

Overall, critical incidents occur most frequently in activities involving patient positioning or transfer (with or without equipment change), cannula change, and endotracheal suction. For the other activities, the data was more evenly distributed. In terms of basic and oral care, AE can occur daily for some caregivers.

#### Q2: nursing activities with the greatest risk of patient harm

The nurses (*n* = 11) were asked to list, as free text, the unexpected events and incidents that pose the greatest risk to the patients. The answers were summarised according to the most frequent occurrences. It was found that tracheostomy decannulation, aspiration, and obstruction of the cannula were considered to be the most risky. The factors that were named as causing tracheostomy decannulation included patient mobilisation and patient delirium.

#### Q3: ranking nursing activities for which the caregiver would like to receive support

The nurses (*n* = 11) were asked to select the most dangerous activities from among 15 nursing activities in descending order. Patient transfer (rank 1: 45%, *n* = 5) was rated as the most dangerous, followed by mobilisation (rank 2: 27%, *n* = 3), transport with equipment change (rank 3: 27%, *n* = 3), positioning of the patient (rank 4: 27%, *n* = 3) and basic care (rank 5: 36%, *n* = 4).

#### Q4: are there any other activities for which you would like to receive support?

Question four asked nurses to describe in free-text format further nursing activities for which they would like to receive support. They stated that they would like to have easier documentation processes to have more time for nursing care. The change intervals for the tube system are not uniformly regulated between the manufacturer and the provider. Standardisation would be desirable in this respect. Decision support with a reminder function for the activities to be carried out with confirmation is desired, as are checklists for ventilation-related activities.

### Analysing causes of adverse events in HMV

**Fig. 3:**
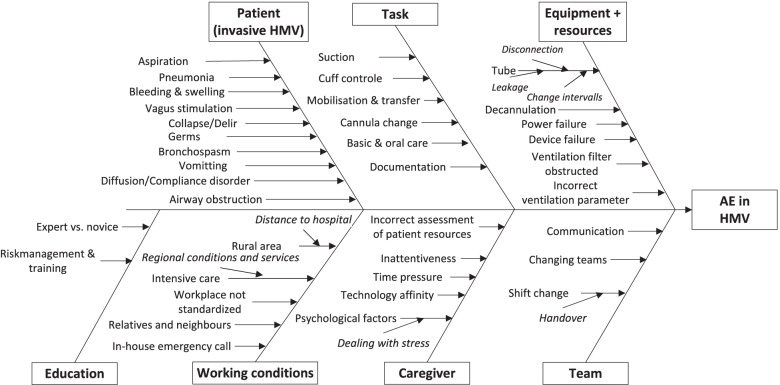
Root-cause diagram (Ishikawa). Contributing factors for AE in the socio-technical system of HMV

The Ishikawa diagram (see Fig. 3) represents a comprehensive modelling of AE in HMV and considers the entire socio-technical system. The horizontal arrow represents the effect or the AE to be avoided. The vertical or oblique arrows are the main influencing factors that lead to the effect. These arrows are divided into categories that have been adapted according to the domain and based on [27]. All data from the previous results has been incorporated into the diagram. All the terms have been checked against, and complemented by, the contents of the “Guidelines for Non-Invasive and Invasive HMV for Treatment of Chronic Respiratory Failure - Update 2017” [5] and the prospective study of [31].

The underlying chronic disease of the patient, such as COPD or a neuromuscular disease, must be considered as one potential cause of AE (see category “Patient”). However, the patient’s state of health can also change because of other acute changes, such as aspiration and airway obstruction, which can contribute to triggering an AE.

The “Task” include the nursing activities that the caregiver performs on the patient. According to the caregivers, all of the tasks mentioned are classified as involving a high degree of risk.

The “Equipment + resources” consists of medical equipment (e.g. the ventilator) and accessories (e.g. the endotracheal tube). This category includes power and device failures, but also tube leakage and tracheostomy decannulation, which was indicated as a particularly dangerous cause of AE.

The “Working conditions” refers to the patient’s home environment and its geographical location. For example, in rural regions lacking in infrastructure, it can take a comparatively long time for an ambulance to arrive in an emergency. Furthermore, each patient’s home environment is set up differently, meaning that standardised care procedures (nurses’ workplace conditions) have to be adapted to each individual patient.

A nurse is usually alone with the patient, but there are also points of contact between nurses. The “Team” category relates to shift change, patient care documentation and handover communication between nurses.

The nurses in the “Caregiver” category are exposed to high stress in medical emergencies. Depending on the complexity of the patient’s care requirements, nurses may also have to perform nursing activities under time pressure.

In the “Education” category, there are aspects that relate to professional education. Work experience, as well as instruction and training are relevant aspects, especially with regard to emergencies.

### Recommendations for risk mitigation

Using the Ishikawa diagram, some of the causes were analysed for risk mitigation. The Table 2 contains the causes from the Ishikawa diagram, the resulting consequence, and the recommended risk management measures. These show that addressing the causes can potentially prevent undesired events from occurring.

**Table 2:** Risk management mitigations

Cause	Consequence	*Target group:* measures for risk mitigation
Mobilisation & Transfer	Failure of ventilation	***Care competence:*** Carers trained to improve fixation of tube ***Care competence:*** Carers trained to recognise signs of medical problems
Airway Obstruction	Failure of ventilation	***Device competence:*** Knowledge about alarm types (high pressure) ***Manufacturers:*** Making alarms understandable ***Medical competence:*** Blood saturation measurement ***Care competence:*** Suction
Tracheostomy decannulation	Failure of ventilation	***Device competence:*** Knowledge about alarm type (low pressure) ***Equipment competence:*** Secure attachment connection ***Manufacturers:*** Mechanisms to avoid tube dragging
Tube Change Interval	Contamination/infection	***Nursing service operator:*** Replacement tube available ***Manufacturers:*** Harmonise change intervals of the tubing material and store them in the device
Power Failure	Device blackout	***Device competence:*** Alarm type (power alarm) ***Nursing service operator:*** Device backup ***Nursing service operator:*** Battery backup Ambu bag Emergency contact numbers
Medical Problem/Health Deterioration	Acute health deterioration	***Environmental conditions:*** Emergency contact numbers ***Environmental conditions:*** Helping system (advice) ***Medical competence:*** Trained to detect early signs of health problems (e.g. silent infection)
General		***Emergency training:*** Training concepts for emergency management ***Nursing service operator:*** Escalation/support concept
		***Nursing service operator:*** Process analysis of the setting and resources at home
		***Nursing service operator:*** Checklist for care process

## Discussion

Adverse events during mechanical ventilation are ubiquitous in hospitals. In a retrospective analysis, 5.6% of patients were affected with a ventilator-associated condition [32]. The CDC has defined objective criteria for ventilator-associated events (VAE) [33, 34]. These are a “deterioration in respiratory status, infection or inflammation, and laboratory evidence of respiratory infection” with guidance for a treatment pathway/algorithm to detect these AE. This data and these outcomes do not exist for care in the home setting. Medical criteria for the detection of pneumonia and infection apply, of course, yet the home setting is characterised by there being a single responsible caregiver. Therefore, our combined data collection methods focused on a subjective assessment of risks in respiratory care. Based on these results, we integrated this data with existing guideline literature and systematic error analysis methods. The result is an root-cause diagram and a list of recommended actions. The recommended actions address the various different actors in the entire socio-technical system. The nursing staff, training staff, manufacturers, and the organisation all have a responsibility to help improve patient safety in HMV. Only multi-causal prevention concepts can help to avoid AE. This root-cause diagram and the recommended actions can be used by manufacturers to improve their equipment and for expanding education concepts for organisations and nursing schools to include the risks mentioned in this study.

The number of causes shows how widespread potential patient harm is. The questionnaire placed a greater focus on the level of harm than the likelihood of occurrence in HMV. In this respect, activities such as ventilation settings, cannula change and patient transport were seen as the main causes of AE. Tracheostomy decannulation, oral care and suction were rated as the most dangerous causes of adverse outcomes. Nurses requested support in patient transfer, mobilisation and positioning of the patient, as well as checklists for nursing activities and reminder functions for ventilator-related activities (e.g. tube change). In the literature, however, AE tend to be associated with medication errors, decubitus and misdiagnosis. In general, the evidence on AE in the home setting is poor [10, 13, 35]. It is estimated that 10% of hospital admissions are due to patient harm that could have been avoided [36]. Approximately four in 10 patients experience harm in an outpatient setting [10]. In [37] it was shown that out of 189 reported events, 39% were due to device error; all other errors were due to caregiver-related causes (e.g. improper device use). Risk mitigation measures are a legal requirement for medical device manufacturers [22], but not for care providers. Nevertheless, care providers should also be able to identify foreseeable risks and take actions and measures to prevent AE [38].

Adverse events do not happen completely unexpectedly. They are subject to a certain degree of statistical distribution and are thus omnipresent. It is only a matter of when and to whom an adverse event will occur. Transparency in the occurrence of AE would give nursing staff the competence to react early to possible causes or to be able to act in emergencies. Therefore, an analysis of the home environment in which a seriously ill person is cared for is essential. Risk management measures must be taken for the care processes and the possible causes, so that AE cannot cause harm.

### Limitations

The limitations of this study are the low number of test persons and the low number of different home care providers and ICUs. Only employees of the project partners took part, which might have resulted in response bias. If a further study were conducted, it would be desirable to interview additional caregivers in order to acquire a broader picture of approaches to addressing adverse events. In question no. 3, participants were not asked about the terms *cannula change*, *device maintenance*, *goose gargle change*, *ventilation filter change*, *inhalation* and *adjustment of ventilation parameters*. Given the absence of these terms, a distorted result is to be expected regarding the lower ranked nursing activities.

## Conclusion

This study has determined that, from the point of view of the nursing staff, there is a need for action to prevent the occurrence of adverse events. Risks in HMV were identified based on expert interviews and questionnaires. The collected data was analysed and systematically mapped onto a root-cause diagram. The grouping of adverse events, risks and hazards resulted in a categorisation to enable the targeted reduction of hazards. For manufacturers, caregivers and care services, the categorisation offers the possibility to expand the list of hazards, to create a checklist for particularly risky care activities, and to develop ideas for innovative decision-making and equipment support.

## Data Availability

We did not record the qualitative interviews, but took notes. These notes are already included in the study. The questionnaire was created and exported with SoSci Survey (SoSci Survey GmbH, Munich, Germany).

## Abbreviations

AE: Adverse Event
HMV: Home mechanical ventilation
ICU: *Intensive Care Unit*
